# Ravines and Sugar Pills: Defending Deceptive Placebo Use

**DOI:** 10.1093/jmp/jhu045

**Published:** 2014-12-10

**Authors:** Jonathan Pugh

**Affiliations:** University of Oxford, Oxford, UK

**Keywords:** *autonomy*, *clinical ethics*, *clinical policy*, *deception*, *placebo*

## Abstract

In this paper, I argue that deceptive placebo use can be morally permissible, on the grounds that the deception involved in the prescription of deceptive placebos can differ in kind to the sorts of deception that undermine personal autonomy. In order to argue this, I shall first delineate two accounts of why deception is inimical to autonomy. On these accounts, deception is understood to be inimical to the deceived agent’s autonomy because it either involves subjugating the deceived agent’s will to another’s authority or because it precludes the agent from acting effectively in pursuit of their ends. I shall argue that providing an agent with false beliefs is not inimical to their autonomy if they are only able to effectively pursue their autonomously chosen ends by virtue of holding those particular false beliefs. Finally, I show that deceptive placebo use need only involve this latter sort of deception.

## I. INTRODUCTION

A recent survey in the United Kingdom has suggested that the majority of UK general practitioners (GPs) have prescribed a placebo at some point in their career (Howick et al., 2013). However, the use of placebos in a clinical context is often deemed to be morally problematic. This is because a great deal of evidence suggests that the placebo effect is strongest when the patient is not aware that the treatment they are receiving is a placebo. As such, in some cases, it seems that physicians can have a *prima facie* moral reason, rooted in the duty of beneficence, to prescribe a placebo deceptively. However, many people object to deceptive placebo use because they claim that it violates the patient’s autonomy.

In this paper, I argue that this latter view is mistaken because it assumes that any sort of deception is inimical to the deceived agent’s autonomy. My aim in this paper is to show that at least one sort of deception can be compatible with respecting autonomy and that the deception involved in certain deceptive placebo use is of this particular kind.

## II. SOME DEFINITIONS AND THE UK STUDY

I begin this investigation by offering a brief explanation of what placebos are, before outlining the moral problem in more detail. Providing an accurate definition of a placebo is *itself* a complex issue, and I shall not enter into this particular debate here; rather, I shall adopt the definitions that Howick et al. (2013) adopt in the survey that I take as the starting point of this investigation. Howick et al. draw a distinction between “pure” and “impure” placebos. On this distinction, pure placebos are interventions that lack any “. . . direct pharmacologically active ingredients for the condition being treated” (Howick et al., 2013, 2). For example, sugar pills and saline injections qualify as pure placebos on this definition. In contrast, Howick et al. define “impure” placebos as:

. . . substances, interventions, or therapeutic methods which have known pharmacological, clinical or physical value for some ailments but lack specific therapeutic effects or value for the condition for which they have been prescribed (Howick et al., 2013, 2).

On this definition, certain pharmacological agents might qualify as impure placebos in certain circumstances; however, many other nonpharmacological interventions will as well. For instance, Howick et al. claim that positive suggestions and unnecessary physical or technical examinations of the patient can all qualify as impure placebos (Howick et al., 2013, 2). For the purposes of this essay, we may understand a fundamental property of any placebo treatment to be that it is an intervention that the physician believes will not have a direct therapeutic effect on the condition for which it has been prescribed.^1^

With this understanding of placebos in mind, we can begin to consider the situations in which they might be used.^2^ Although there is some debate regarding the efficacy of placebos (Hróbjartsson and Gøtzsche, 2001),^3^ it is generally agreed that placebos can be used to ameliorate various unpleasant psychological symptoms that can accompany somatic and psychiatric illness, such as pain, anxiety, and depression (Turner et al., 1994; Schweizer and Rickels, 1997; Kirsch and Sapirstein, 1999; Foddy, 2009a). As such, it has been claimed that placebos can have a therapeutic effect on patients who are suffering from a wide array of medical conditions, ranging from psychiatric conditions such as clinical depression (Kirsch and Sapirstein, 1999) to somatic conditions such as multiple sclerosis (La Mantia et al., 1996) and Parkinson’s disease (Goetz et al., 2008).

Of course, it will often be the case that a physician is able to recommend an intervention that they believe *will* have a direct biological or pharmacological effect on the patient’s condition; we might term such therapies “active therapies.” One example of an active medication would be the prescription of antibiotics for a bacterial infection. In prescribing this medication, the physician is prescribing a drug that he is justified in believing will ameliorate the patient’s condition by virtue of a pharmacological mechanism that can be said to have a direct ameliorating effect on the patient’s condition, insofar as the drug, if efficacious, will destroy the bacteria responsible for causing the patient’s symptoms.

However, as Foddy illustrates, there are a number of situations in which a placebo, rather than an active therapy, will be the best, or indeed *only*, available treatment for a particular condition (Foddy, 2009a). This may be the case for a number of reasons. First, there may just not be an available active medication for the patient’s condition (or the patient may have refused available active medications). For example, Foddy lists irritable bowel syndrome as a currently untreatable condition for which deceptive placebos are equivalent to the best available treatment (Foddy, 2009a, 5). Second, available active medications may not be any more therapeutically effective than a placebo and yet put the patient at risk of serious side effects (which the placebo will not). For example, suppose that a patient goes to his GP complaining of sleeping problems. He tells his GP that he has undergone cognitive behavioral therapy but that this has not helped; as such he asks to be prescribed sleeping pills. Although the GP believes that sleeping pills would help, she is also aware that the sleeping pills that the patient has requested carry a high risk of addiction, as well as other adverse side effects. In this case, the physician might believe that it would be best to prescribe either a pure or impure placebo if she believed that it would ameliorate the patient’s sleeping problems without subjecting him to the risk of addiction and other adverse side effects.^4^

Another case in which a physician might feel that it would be best to prescribe a placebo is when he is unable to ascertain what is causing the patient’s discomfort, and he believes that a placebo might help to alleviate the discomfort without subjecting the patient to the side effects of active medications.^5^ Finally, Kolber also points out that placebos have a high diagnostic value in distinguishing between those patients who suffer from epilepsy and those who suffer from pseudoseizures (Kolber, 2007).

The ethical problem with the clinical use of placebos arises because empirical evidence suggests that placebos are most efficacious when the patient is not aware that the treatment being received is merely a placebo (Foddy, 2009a, 7). Although this empirical point is not universally agreed upon (Bostick et al., 2008; Kaptchuk et al., 2010), I shall assume that it is true for the purposes of this paper, on the basis of the empirical evidence discussed by Foddy, as well as philosophical considerations in its favor highlighted by Groll (2011). However, it should be acknowledged that my conclusions regarding the moral permissibility of deceptive placebo use are conditional on this empirical point.

Although the exact mechanism underlying the placebo effect is widely debated, a considerable amount of evidence suggests that in order for placebos to be fully effective, the patient must have some expectation that the treatment being received will benefit him (Foddy, 2009a, 9). As such, informing the patient of the fact that his treatment is an inert placebo (which would render the treatment a “revealed placebo”) may in fact undermine the placebo’s therapeutic power, since the revelation will often undermine the patient’s expectation that he will benefit from the treatment. Accordingly, in order to maximize the therapeutic effect of a placebo, it seems that physicians must engage in a degree of deception so as to obscure the fact that the treatment they are prescribing to their patient is an inert placebo. Indeed, in view of the questionable efficacy of revealed placebo use, Foddy goes so far as to claim, somewhat provocatively, that “[i]f placebos are to be used in the clinic at all, they ought to be used deceptively” (Foddy, 2009a, 7).

If this is right, then the use of placebos in clinical practice raises an ethical problem, since, if the physician believes that a placebo is the best available treatment for the patient, then the physician’s duty of beneficence seems to require that the patient be deceived. Yet we are loath to claim that it is morally permissible, let alone morally required, for physicians to deceive their patients; such an assertion smacks of paternalism and seems to be an affront to the value we place on patient autonomy. In view of this, the American Medical Association explicitly prohibits deceptive placebo use (Bostick et al., 2008). Moreover, although the General Medical Council in the United Kingdom does not explicitly prohibit deceptive placebo use, the council’s guidelines advise that physicians should always be open with patients about the treatments that they are prescribing in a manner that seems to preclude deceptive placebo use (General Medical Council, 2013).

Even if one does not agree that deceptive placebo use has significantly more therapeutic power than revealed placebo use, data from a recent survey of GPs in the United Kingdom suggest that there is still an ethical problem regarding deceptive placebo use, since the vast majority of UK GPs seems to be using placebos in a somewhat deceptive manner.^6^ Howick et al. found that 97% of their respondents had used impure placebos at least once in their career, whereas 12% of them had used pure placebos (Howick et al., 2013). As such, the study shows that the use of placebos is prevalent in UK medical care. These figures are consistent with other international surveys; a recent meta-analysis of GP placebo use survey across 12 countries found that 17% to 80% of practitioners had used “pure” placebos at least once in their career and between 54% and 57% had used impure placebos (Fässler et al., 2010).

However, an equally interesting finding from the UK study concerned GP beliefs about the ethical acceptability of placebo use. Although placebo use is prevalent, Howick et al. (2013) found that around 82% of their respondents believed that the use of placebos^7^ is not ethically acceptable when it involves deception. This might lead one to think that the majority of the large number of GPs using placebos must be revealing that fact to their patients; but the evidence suggests that the opposite is the case. In the case of impure placebos, only 8% of the respondents who had used placebos told their patients that the treatment they were receiving was an impure placebo, whereas 9% told their patients the exact nature of their treatment in the case of using pure placebos (Howick et al., 2013, 3–4).

The key to explaining this apparent inconsistency is suggested by the fact that around half of the respondents who prescribed either sort of placebo provided patients with a *partial* disclosure about the treatment they were receiving, telling them something like “this therapy has helped many other patients” (Howick et al., 2013, 3–4). This suggests that, although the GPs in this survey believed that deception was not ethically acceptable, they had a robust conception of deception in mind, according to which merely omitting to reveal certain information about the nature of a treatment does not amount to deception.

However, such a view of deception is inadequate; although the partial disclosure that half of the respondents gave to their patients was true, this does not entail that it was not deceptive.^8^ To see why, it is crucial to acknowledge two things. First, it seems plausible to assume that many patients are likely to infer from their GP’s partial disclosure that the therapy they are receiving has previously helped other patients *because it is an active therapy*. After all, it seems plausible to posit that many patients who lack medical expertise are likely to believe that a therapy will only have been able to help other patients if there was something in the therapy *itself* that caused the other patients to get better; that is, many patients will only expect to benefit from a therapy if they believe that it is an active treatment.

Second, it seems that the most plausible explanation of *why* many physicians provide only a partial disclosure about the nature of placebo therapies is that they want to conceal the true nature of the therapy so that the patient will not realize that the treatment is inert. Of course, in other treatment contexts, there could be other reasons why a physician might provide only a partial disclosure concerning the nature of a treatment to the patient. For example, in some cases, a full disclosure might cause disproportionate psychological distress, or the physician might not believe that the patient would be able to understand a full disclosure. However, it seems that many of the salient reasons that are sometimes offered to justify a partial (rather than full) disclosure will not apply in the case of a placebo treatment; for instance, since the placebo is inert, a full disclosure about the nature of the placebo is unlikely to cause the patient disproportionate psychological distress, and it is likely that many patients would be capable of understanding a full disclosure concerning the nature of the treatment. Accordingly, it seems that the best explanation of why GPs provide only a partial disclosure to their patients in this context is that they believe that the placebo will be most efficacious if the patient is not fully aware of its nature. Indeed, they may even want their patient to make the reasonable, but incorrect, inference (from their partial disclosure) that the therapy is an active treatment, since, as I suggested above, it seems plausible to suppose that many patients will only expect to benefit from *active* treatments.^9^

Therefore, although physicians do not lie to the patient in simply saying that the therapy they are offering has helped others, it seems that the most plausible explanation of why they limit their disclosures to this partial description is that they want to mislead their patient into making a rational inference to the (false) belief that the treatment being received is active; the physician who does this will do so in the belief that the placebo will be most efficacious if the patient holds this false belief.

With this understanding of deception in mind, we might say that Howick et al.’s (2013) data show not only that deceptive placebo use is widespread in UK medicine but also that it seems likely that some physicians are, in a sense, *self-deceiving* about the nature of their placebo use; although the majority of them claim that it is not ethically acceptable to use placebos deceptively, Howick et al.’s data suggest that it is likely that some of the GPs who use placebos are doing so in a manner that may appropriately be deemed deceptive. As I suggested above, physicians who provide partial disclosures about their placebo use may be understood as deceiving their patients if we make the plausible assumption that some patients are likely to infer from the physician’s partial disclosure that they are receiving an active, rather than inert treatment, and that the physician’s reason for providing only a partial disclosure is that he wants patients to make this incorrect inference because he believes that this will make the treatment more effective.

In view of this, it might seem that there is something of a moral crisis in UK medicine; if deception is as widespread as the data suggest, what hope is there for patient autonomy? In the remainder of this paper, I argue that the prevalence of deceptive placebo use is not the threat to patient autonomy that many claim it to be. In order to begin making this argument, we must first consider why deception is thought to undermine autonomy. It is to this issue that I now turn.

## III. TRUE BELIEFS AND AUTONOMOUS AGENCY

The concept of autonomy is, as Dworkin points out, something of a “term of art” (Dworkin, 1988, 7) in philosophy, and it is the subject of myriad interpretations. I shall not rehearse these various interpretations here; all that we need acknowledge is that the concept of autonomy broadly aims to capture something like the property of self-government. In practical ethics, we often understand this to mean that the autonomous agent is, *inter alia*, one who is able to direct his own conduct, and who is accordingly able to express himself through his actions; by implication, the autonomous agent is one whose will is not subjugated to determining forces of the sort that may be deemed to undermine his ability to direct his own conduct.

Even with this rudimentary understanding of autonomy in mind, we can begin to consider two prominent accounts of why deception is thought to undermine autonomy and why true beliefs are often important to autonomous agency.

### Taylor’s Account

According to Taylor, deception is inimical to autonomy because, in cases of deception, *the deceived agent’s will is subjugated to the deceiver’s*; the deceiver controls the deceived in a manner that is incompatible with the latter’s self-government because the nature of the deceiver’s controlling influence means that the deceived agent is no longer able to direct his conduct in the light of his own desires and values (Taylor, 2009). As an illustration of this, Taylor offers the example of Iago’s control over the eponymous hero Othello in the famous Shakespearean tragedy (Taylor, 2009, 4).

Taylor’s account is summed up in his threshold condition: In order to be autonomous with respect to a practical decision, an agent must (*inter alia*) meet the following condition:

*Threshold Condition*: If the information upon which the agent bases her decision has been affected by another agent with the end of leading her to make a particular decision . . . and if she is not aware of the way in which this info has been affected *then* she did not make the decision that (her deceiver) intended her to make (Taylor, 2009, 7).

### Killmister’s Account

In her analysis of autonomy and false beliefs, Killmister suggests (*pace* Taylor) that agents may lack autonomy when they act on the basis of certain false beliefs even if they have *not* been intentionally deceived into having them. Consider the following example discussed by Killmister:

Let’s say I form the intention to eat a piece of cake. To fulfill this intention, I go to what I take to be the fridge and retrieve what I take to be a delicious cream-filled Swiss roll. In fact, what I have done is gone to the cupboard and retrieved a roll of dog food, which I am eagerly bearing towards my mouth (Killmister, 2013, 521).

Although there is no intentional deception involved here, Killmister suggests that the agent in this example would not be acting autonomously in eating the dog food because the false belief ensures that the action cannot satisfy the intention. As Killmister puts it:

The order I give myself is to satisfy my desire for cake, yet that is not possible given the action I perform. As such, the action was not under the direction of my will (Killmister, 2013, 521).

The point that Kilmister is making in this example is that although autonomy demands that agents decide how to act in the light of their own desires and values, this is not sufficient for their being autonomous in their conduct. On Killmister’s account, autonomy is understood as also requiring a degree of personal efficacy; if agents are to be autonomous, they cannot be precluded from acting effectively in pursuit of their ends by their false beliefs. Therefore, on Killmister’s account, deception can serve to undermine autonomy because deception can prevent agents from holding true beliefs that will often (but I shall argue below not always) be essential to one’s ability to act effectively in pursuit of one’s ends.

There is of course more to say on the relationship between autonomy and true beliefs. However, I have said enough here to elucidate two key ways in which deception may be understood to undermine autonomy. With this in mind, in the next section, I shall consider the practical application of these theories of deception and autonomy and explain why deceptive placebo use need not be inimical to the patient’s autonomy.

## IV. RAVINES AND SUGAR PILLS: THREE SORTS OF DECEPTION

To begin my argument in favor of deceptive placebo use, I appeal to an analogy to elucidate certain ways in which Killmister’s and Taylor’s theories can be applied. I then consider a case that is analogous to deceptive placebo use and in which I believe it is plausible to claim that deception does not undermine the deceived agent’s autonomy. I employ this strategy because it is useful to first consider the question of when deception undermines autonomy in isolation from the prevailing norms that are understood to govern ethical medical conduct, such as the norm that doctors ought not to deceive their patients. Only by first considering this issue in isolation from the ethical norms that govern the profession of medicine will I be suitably placed to question some of those very norms later in the paper. Finally, although there are some disanalogies between deceptive placebo use and the third case that I present here, I highlight and discuss these below.

With this in mind, consider this scenario:^10^

Jim and Becky are being pursued by a beast deep in the jungle and running for their lives. Jim is some way ahead of Becky and comes to a ravine; he realises that they *must* somehow get across it to escape. Jim believes that he can leap distances of up to 5 metres, but he knows that Becky believes that she is not capable of jumping anything over 4 metres. However, Jim believes that Becky is just being irrational; he knows that she could jump the same distances as he can. However, she is only able to do so if she *believes* that the jump is at most 4 metres.

Now let us imagine one permutation of this scenario.

*Case One*: Suppose that Jim calculates the ravine as being 3 metres wide. Suppose further that Jim really dislikes Becky; as such, he calls her on her walkie-talkie and leads her to adopt the false belief that the only way to escape the beast is by jumping a 6 metre ravine. Becky believes Jim and refuses to even attempt the forthcoming jump, so Jim says that the best thing that she can do is try and fight the beast off, even though they know that this will be futile.

Both Taylor and Killmister would agree that Jim deceives Becky here in a manner that is inimical to her autonomy. On Taylor’s account, Jim’s deception undermines Becky’s autonomy because, by influencing her to adopt a false belief, he subjugates Becky’s will to his own; Jim exerts control over her decision about what to do so that her decision will reflect his ends rather than her own. Alternatively, Killmister might claim that Jim’s deception here undermines Becky’s autonomy because it ensures that she will be informationally cut off from achieving the end of saving her own life. Either way, the fact that Jim directs Becky’s decision-making process through his deception seems to render her heteronomous.

This sort of autonomy undermining deception can occur in a clinical context, and when it does we often find it abhorrent. For example, we can imagine a physician deceiving a patient into thinking that a procedure was not at all risky in order to obtain consent to participate in a clinical trial that will further the physician’s career. This would be a paramount example of a way in which a physician can deceive the patient in a manner that undermines the latter’s autonomy. Similarly, in the context of placebo use, suppose that a doctor prescribed a placebo deceptively, not because she thought that the placebo would help ameliorate the patient’s condition but rather because she wants to get rid of an annoying patient quickly. Again, it seems clear that such deception undermines the patient’s autonomy.

Consider now a different permutation of the ravine scenario:

*Case Two*: Suppose this time that Jim and Becky are good friends. Jim calculates the ravine as being 3 metres wide, and he believes that Becky and he can both easily clear it. However, suppose that he has misjudged the distance; the ravine is in fact 4.5 metres wide. He calls Becky and tells her that they will need to jump a 3 metre ravine in order to escape. Becky hears all this, and prepares herself for a 3 metre leap and fails to clear the ravine.

On Taylor’s account, Becky is autonomous with respect to the decision that leads her to jump only 3 m, since she was not *intentionally* deceived by Jim. In contrast, on Killmister’s account, Becky lacks autonomy with regard to her act, because she lacks control over the success of her efforts to achieve her ends by virtue of her poor informational condition. This suggests a different way in which deception might undermine autonomy. In this case, it seems inappropriate to claim that Becky is heteronomous, since Jim is not providing her with false beliefs in order to direct her decisions to his own ends and thereby subjugate her will to his. Rather, if we endorse Killmister’s picture, we may simply say that Jim’s testimony renders Becky nonautonomous.

Again, this sort of autonomy undermining deception could occur in a clinical context with placebos. Suppose that a patient came to the physician with a life-threatening condition requiring an immediate active intervention that was readily available. However, suppose that the physician failed to reveal to the patient that she had no expertise in the area of medicine that was central to the patient’s condition and instead prescribed a deceptive placebo, believing that this would cure the patient, despite the fact that the placebo would have a negligible effect on the patient’s condition. Here, it seems that the physician’s deception will ensure that the patient is informationally cut off from successfully achieving the end of better health.

Consider now a final permutation of the ravine scenario.

*Case Three*: Suppose that this time the ravine is wider; Jim, correctly this time, calculates it as being 5 metres wide. He knows that in their state of high adrenalin, both he and Becky can jump this distance. However, he also knows that Becky will fail to make the jump if she knows that the ravine is 5 metres wide; her fear will hold her back. So he calls Becky and tells her that the only way to escape is to jump a ravine that is 4 metres wide. Since he knows that this is at the limit of the distances that Becky believes she can jump, Jim has good grounds for believing that she will put all her effort into it, and leap *at least* 5 metres, even though she herself believes that the ravine is only 4 metres wide. Becky successfully clears the ravine.

Does Jim’s deception undermine Becky’s autonomy? Consider this case first on Killmister’s account. Although Becky acts on the basis of a false belief, it does not seem that she is informationally cut off from achieving her end. Quite the contrary; her holding this false belief is *necessary* for her success. As I explained in the previous section, agents normally need to have true beliefs concerning the nature of the means to their autonomously desired ends if they are to be efficacious in pursuing those ends. However, this case is extraordinary insofar as Becky will *only* be able to be efficacious in this way if she holds a very specific false belief about the means to achieving her end; namely, the belief that the ravine she has to jump is only 4 m wide.

There is perhaps one sense in which Becky’s autonomy could be undermined here on Killmister’s account. Suppose that one of Becky’s goals is to hold as many true beliefs as possible; Jim’s deception would surely undermine the pursuit of this goal. However, before claiming that Jim’s deception would therefore render Becky nonautonomous, we must remember that Becky’s achievement of this goal and her achieving her goal of surviving are *mutually exclusive*; moreover, by virtue of the circumstances, it is *Jim*, not Becky, who must decide which of her goals to honor. In some sense, then, Becky’s autonomy has already been diminished by the nature of her situation, since it is Jim who must decide which information she receives, and, in so doing, which of her goals to prioritize. However, given this situation, Jim is surely justified in believing that Becky prioritizes her continued survival over the value she places on holding true beliefs; in fact, the latter is obviously integral to her *global* autonomy. Accordingly, insofar as the false belief that Jim leads Becky to have is essential for Becky’s effective pursuit of an autonomously chosen end that Jim is justified in believing Becky would prioritize over having true beliefs, I claim that Jim’s deception can be regarded as facilitating Becky’s autonomy, all things considered.

In contrast, on Taylor’s account, Jim’s deception *must* undermine Becky’s autonomy. If Becky decides to jump, then she will fail to meet Taylor’s threshold condition. After all, Jim clearly affects the information on which Becky bases her decision, with the end of leading her to make a particular decision; furthermore, she is not aware of the way in which he has affected the information.

In my view, this just goes to show that Taylor’s condition is incomplete in its current form. Its flaw lies in the fact that it fails to distinguish between deceptions that are intended to subjugate the deceived agent’s will to the deceiver’s (as in case one) and deceptions that are intended to facilitate the deceived agent’s pursuit of her own autonomously chosen ends (as is the case here). In case one, it is clear how the deception undermines autonomy, since the deception serves to replace Becky’s ends for Jim’s in Becky’s practical deliberations. However, in case three, the deceived agent’s ends are left intact; Jim’s deception does not alter the end which Becky’s acts are conducive to achieving. Moreover, the false belief Jim persuades Becky to adopt is *necessary* for her successfully achieving her end.

It might be argued that Jim does subjugate Becky’s will here, because although the deception promotes *her* end, the deception involves an imposition of a certain means to that end that Becky would not accept. Becky thus has no *local* autonomy with respect to the means that she takes to her end; she does not decide how she will go about achieving her end of survival. Again, I think that this point must be conceded; however, I do not believe that this means that Becky therefore lacks autonomy, at least not the autonomy that is of importance to us. This is because although Jim’s deception might undermine her local autonomy with respect to her decision about the *means* she takes to the end of her own survival, it does not undermine Becky’s autonomy with respect to this *global* commitment.

The important thing to acknowledge here is that it seems implausible to claim that having local autonomy with regard to a decision about the means one uses to pursue one’s ends is more important than preserving one’s ability to successfully pursue that global commitment at all. Indeed, we often sacrifice our local autonomy with regard to our decisions about the means that we will use to achieve our ends, when doing so will help facilitate our successful pursuit of those global commitments and when the means that we take do not instantiate other important values. For example, we might ask a financial advisor to tell us which pension policy is most likely to give us the money we need in retirement rather than make the decision independently.

Having said this, if there were several different means that the agent could use to achieve their ends, and if any of them could be used to effectively pursue it, then the agent’s choice of means might itself plausibly be deemed to be an expression of their autonomy, since the means might also instantiate other values that it should be up to the agent to decide how to weigh. However, this is a very different case from the one that I am describing in case three, where a third party must choose to honor either what they are justified in assuming is the agent’s all-things-considered most salient global commitment, while sacrificing her local autonomy with respect to the means she takes to achieving that end, or to safeguard her local autonomy and to prevent her from acting effectively in pursuit of her global commitment.

Accordingly, I believe that case three is an example of intentional deception that does not undermine the deceived agent’s autonomy. I suggest that the deception involved in case three does not undermine the deceived agent’s autonomy because of two key features of the case. First, the deception does not involve Jim subjugating Becky’s will to his own, insofar as the deception serves to further an end of Becky’s that Jim justifiably believes would take priority in her practical deliberations, rather than an end of his own. Second, in order for Becky to act effectively in pursuit of this end, it is *necessary* that she holds the false belief that she has been led to have by Jim.

Crucially, it seems that the deception involved in certain instances of deceptive placebo use is broadly analogous to the deception involved in case three. As Kolber points out, it seems plausible to claim that patients “. . . seek medical care first and foremost to feel better” (Kolber, 2007, 115); the end that they seek to pursue is their own health and its contribution to their well-being. As I suggested in the second section of this paper, there are some cases in which a deceptive placebo is the *only* way in which a GP can help to remedy a patient’s ailment. If a GP prescribes a deceptive placebo in these cases, she does not seem to be subjugating the patient’s will; she is deceiving the patient in order to facilitate the achievement of the patient’s end, not her own. Furthermore, the patient’s falsely believing that his placebo treatment is active will, in these cases, be necessary for the treatment’s therapeutic effect.

There are of course some important disanalogies between the third ravine case and such deceptive placebo use. In the ravine case, the deception saves Becky’s life, and this will hardly ever be the case in deceptive placebo use. Of course, the ravine case could be changed to reflect this; suppose that Becky is not being chased by a beast, but will instead experience significant distress if she falls into the ravine, or stays on the side where she is currently, a degree of distress similar to that experienced by those who suffer serious side effects of certain active medications or of the symptoms that the placebo could serve to alleviate.

This would somewhat weaken the intuitive pull of the analogy. Indeed, some might argue that although Jim is justified in assuming that Becky would prefer her own survival to holding true beliefs about the ravine, doctors are not warranted in assuming that patients would prefer to feel better than to have true beliefs about the treatment they are receiving. Hester and Talisse raise this objection to Foddy’s defense of deceptive placebo use; they point out that “. . . a patient might rightfully value knowing the truth about his/her condition over the benefit he/she might receive from a placebo” (Hester and Talisse, 2009, 23).

Foddy responds to this charge by questioning whether a reasonable patient could in fact prioritize the value of truth over the value of health in this way (Foddy, 2009b). Kolber, however, raises one counterexample in which this could be the case involving religious believers whose beliefs forbid them from consuming inert medications (Kolber, 2007). There are also other examples in which a patient might feasibly prefer to have true beliefs about his condition over the amelioration of some of its unpleasant symptoms. For example, suppose that the patient is only experiencing minor suffering as a result of his symptoms or that the condition that the physician is hoping to ameliorate by administering a placebo is a chronic, rather than acute, condition.

A more powerful response to Hester and Talisse’s objection is to point out that when a placebo is the *only* available treatment, then the physician is bound by the nature of the situation to be assuming the patient’s values *whatever she does*.^11^ If she refrains from prescribing a deceptive placebo, she assumes that the patient values having true beliefs about his condition over the amelioration of his symptoms. Moreover, assuming that revealed placebos are less efficacious than deceptive placebos, telling the patient about the nature of the treatment would make a similar assumption. Thus, the assumption of the patient’s values here is unavoidable. Accordingly, it seems that in many of these cases, the physician could plausibly have justifiable grounds for assuming that the patient would give greater weight to the amelioration of his symptoms than he would to the value of being free from deception.

What might justify a physician in believing this? Intuitively, it seems that two factors will be central to answering this question. The first is what the physician knows about the patient’s attitudes toward deception; if the physician has known the patient for many years, this may allow her some insight into how that patient would feel about his being deceived in order to ameliorate his condition. The second is the severity of the patient’s symptoms; the more pain that the patient is in, the more plausible it seems for the doctor to believe that the patient would have a stronger preference for having his pain alleviated than for not being deceived. Moreover, it does not seem that the patient’s condition needs to be life-threatening in order for the physician to justifiably believe that the patient would prefer the amelioration of his condition to not being deceived about his medication. For instance, consider a patient suffering from irritable bowel syndrome who complains that his condition causes him significant pain and prevents him from enjoying a social life; here, I suggest, it seems plausible that the doctor might be justified in believing that the patient would prefer the amelioration of his condition to having true beliefs.

Of course, there will borderline cases in which it is not clear whether the patient’s symptoms are severe enough to justify the physician’s belief. In such cases, one illuminating question that physicians can ask themselves is “to what extent is the patient’s condition restricting his ability to effectively pursue his own autonomously chosen goals?” Since the physician is deciding whether or not she can justifiably override the patient’s *local* autonomy by deceiving him, it seems that the extent to which deceiving him will facilitate his *global* autonomy should be integral to an assessment of whether that course of action is justifiable. My own intuition in the irritable bowel syndrome case is that the extent to which the patient’s condition is hindering his pursuit of his own autonomously chosen goals is such that the physician would be justified in believing that the patient would prefer to have his condition ameliorated over having true beliefs.

Furthermore, even though there might be cases in which patients might feasibly prefer to have true beliefs about their condition to having it ameliorated, rather than appealing to these cases in order to claim that physicians ought to be prevented from prescribing deceptive placebos to *anybody*, these cases suggest that the physician’s decision about whether to prescribe a placebo should be taken on a case-by-case basis. In order for placebo use to be morally justified, the physician must be justified in deeming placebo use to be necessary for the patient’s amelioration and justified in believing that the patient will place greater value on the amelioration of his condition than he will on knowing the truth about his treatment.^12^ In my view it would be far better if we felt comfortable trusting our physicians to make a professional judgment in these cases, rather than placing a wholesale ban on deceptive placebo use, and with it, sacrificing the therapeutic benefit of placebos.

Having said this, it may be objected that physicians are fallible, and they may sometimes make the wrong call about when it is appropriate to use placebos.^13^ In view of this, the question of whether to allow deceptive placebo use perhaps becomes a question of justice rather than autonomy. Is it worse to deny the therapeutic value of placebos wholesale in order to prevent giving some patients placebos that do not want them? The answer to this, I believe, will depend largely on the numbers in question; I therefore join Barnhill in claiming that in order to provide a full analysis of the morality of deceptive placebo use, we need more data on what patient’s actual attitudes to placebos are (Barnhill, 2011, 22).^14^

An alternative compromise position might be to get physicians to ask their patients about whether they are happy to receive a deceptive placebo prior to a medical consultation. However, as I have already explained, the efficacy of placebos seems to depend on their being administered deceptively. It might be argued that this problem could be circumvented by asking patients to sign a consent form before *any* treatment declaring that they are happy to receive a placebo deceptively if the doctor believes that such measures are necessary. However, although such a strategy seems promising, Kolber has astutely pointed out that it could have an unintended damaging effect on the efficacy of placebos; as Kolber suggests, if patients believe that *any* treatment that they receive has a good chance of being a placebo, then this is likely to diminish their expectation of benefit from the treatments they receive (Kolber, 2007). Crucially, this would also impact on the efficacy of *active* medications, insofar as even active medications rely in part on expectation of benefit for their therapeutic power. With placebos, then, it seems that we cannot have our cake and eat it, too.

To return to the position that I am defending here, I am not advocating a blanket acceptance of all deceptive placebo use; there will be some cases of deceptive placebo use that are disanalogous to case three and that would serve to undermine the patient’s autonomy. A clear instance of this would be if the physician gave a placebo deceptively in order to get rid of an annoying patient. For deceptive placebo use to be ethically acceptable, it is crucial that the patient’s ends are foremost in the prescribing physician’s mind. Moreover, the physician should consider whether the placebo might cause other deleterious effects^15^; for example, if the physician has grounds for believing that the patient will react badly to a placebo, then she ought not to prescribe it, since it will no longer be in the patient’s best interests.^16^

It is also important that the placebo treatment is *necessary*. If alternative treatments are available, then deceptive placebo use might undermine patient autonomy because the different available options may not just represent means to the end of health; they may also instantiate other values that the patient takes to be of importance. As such, the deception robs the patient of choice about which values to prioritize, and this *does* seem integral to his autonomy. If this is right, it might be claimed that ethical deceptive placebo use ought to be restricted to cases in which a placebo is the *only* available treatment.^17^

As such, I claim that it is morally permissible for GPs to prescribe deceptive placebos if they adhere to two ethical guidelines. First, the GP must prescribe the placebo with the intention of promoting her patient’s ends rather than her own, and she must be justified in holding the belief that the patient would value the amelioration of his condition over holding true beliefs about his treatment. Second, the prescribing physician must be justified in believing that a placebo is a necessary means of promoting the patient’s health.

In response to my arguments, it might be objected that permitting deceptive placebo use may be the beginning of a slippery slope, which will lead to widespread deception in health care and which will ultimately undermine the relationship of trust between patients and their physicians.^18^ I do not find this objection compelling; I believe that it is unlikely that deceptive placebo use carried out in accordance with the ethical guidelines set out here would lead to such a slippery slope because such deceptive placebo involves a unique kind of deception, different in kind to the sorts of deception that we find morally problematic. The reason that this sort of deception is almost unique in health care is not merely because the physician carries out the deception in order to further the patient’s own ends (one can easily imagine other, more questionable forms of deception, which were similarly motivated); rather, it is because the patient’s holding the false belief that he is deceived into having is *necessary* for his amelioration, since the patient will only recover if he believes that his treatment is active. As such, this deception can be set apart as different in kind to the sorts of deception that would serve to undermine trust.

## V. CONCLUSION

I have provided a philosophical defense of deceptive placebo use by providing an explanation for why the deception involved in certain uses of deceptive placebos is not inimical to autonomy. Of course, this argument by itself does not mean that health policy ought to allow physicians to prescribe placebos deceptively. There are practical reasons why such a policy might be problematic. First, despite the arguments that I have made above concerning the unique nature of the deception involved in ethical deceptive placebo use, the public are likely to be suspicious of a policy allowing physicians to be deceptive in any way; in order for such a policy to have widespread acceptance, it seems that there would need to be widespread education about the nature of placebos and an understanding of why the deception involved in their use is unlike deception of the sort that undermines autonomy.

However, the bigger problem, as Kolber points out, and to which I alluded above, is that such a policy could have an unintended damaging effect on the efficacy of placebos. If policy allowing deceptive placebo use served to engender a widespread suspicion of all therapeutic interventions, then we have a good reason to avoid such a policy. In conclusion then, perhaps deceptive placebo use needs to remain impermissible from the perspective of policy in order to preserve the covert nature that contributes to its therapeutic power, a therapeutic power that Howick et al.’s study suggests UK GPs at least are taking advantage of. My argument in this paper has been that they do not necessarily violate the autonomy of their patients in doing so if they are doing so in accordance with the ethical guidelines that I have suggested here, given the unique nature of the deception involved in deceptive placebo use that adheres to these guidelines.

## NOTES

1. This raises the question of what “direct” is meant to connote here. We might say that a treatment has a direct effect on a condition if there is some physiological or pharmacological mechanism that at least partly explains the treatment’s efficacy. Groll presents an alternative understanding, in which he defines placebos as treatments operating through a causal cognitive mechanism and nonplacebos as those operating through a causal noncognitive mechanism. See Groll (2011).

2. In the following paragraphs, I shall draw heavily from Foddy’s comprehensive discussion of the debate concerning the efficacy of placebos. For a far more extensive discussion of these issues, see Foddy (2009a).

3. Once again, I direct the interested reader to Foddy’s comprehensive discussion of these issues. Foddy (2009a, 4–5).

4. In these cases, I am assuming that the physician has taken all reasonable steps to investigate the possibility of other treatments for the patient’s condition. It should be acknowledged that a wide range of therapies that do not carry risk of serious side effects have been used to try and combat insomnia (such as acupuncture, for example). However, although these therapies may be effective in some cases, there is no substantial empirical proof that any of these therapies have a *direct* effect on insomnia. Accordingly, it seems plausible that a physician could quite justifiably believe that these alternative therapies are impure placebos. In such a scenario, if the physician were to prescribe one of these therapies, then this would still count as her prescribing a placebo, insofar as she herself believes that the therapy she is prescribing will only ameliorate the patient’s condition by virtue of the placebo effect.

5. In this case I am assuming that the physician is justified in believing that no reasonable further steps can be taken to ascertain the nature of the patient’s condition.

6. It should be acknowledged that my conclusions in this section are contingent on the claim that the respondents to Howick’s survey invoked the same definitions as the authors of the study delineated in the survey.

7. Slightly more (82.4%) respondents found the deceptive use of impure placebos unacceptable than those who found the deceptive use of pure placebos unacceptable (82.0%) (Howick et al., 2013).

8. See also Barnhill (2011, 22). Some ethicists have argued that it is worse to get someone to adopt a false belief by intentionally providing them with false information than it is to do so by intentionally framing the (true) information that you give them in misleading ways (Jackson, 1991; Gillon, 1993; Benn, 2001). However, even those who claim this agree that both of these methods amount to deception.

9. An anonymous referee pointed out that patients need not necessarily make this inference and may instead infer only that they will be helped by the treatment. Although this may be right, this does not mean that the partial disclosure itself is not deceptive. The fact that some patients may not make the further inference that the therapy is an active treatment does not change the fact that, in providing only a partial disclosure, the physician is choosing to omit key information about the nature of the treatment from her disclosure, in order to conceal the true nature of the treatment from the patient, so that the patient will not realize that the treatment is inert.

10. Foddy considers some analogous cases. See Foddy (2011).

11.
Savulescu (1995) suggests that physicians face this problem far more generally.

12. As an anonymous reviewer highlighted, in the normal case, a patient’s autonomy is best served by a physician discussing different treatment options with the patient and reaching a joint decision. I agree that this is normally the case; moreover, I agree that in the cases when they use deceptive placebos, physicians should attempt to elicit the patient’s values without revealing the fact that they are considering prescribing a placebo and make their treatment judgment on the basis of that discussion. However, in the cases in which I suggest deceptive placebo use is morally permissible, my argument is that discussing the patient’s treatment options (rather than his values) will not enhance his autonomy in this unique case. On the assumption that revealed placebos are less efficacious than nonrevealed placebos, then the physician cannot enter into a dialogue about the patient’s treatment options without taking away the treatment option that is most efficacious (namely, the nonrevealed placebo).

13. An anonymous reviewer pointed out to me that if the physician’s justification for prescribing a placebo deceptively is that she is unable to ascertain what is causing the patient’s discomfort (and believes that a placebo might help to alleviate the discomfort without subjecting the patient to the side effects of active medications), then this may not be a sufficient moral justification, since a patient may want to know that his doctor does not know what else to do so that he can get a second opinion. As such, if a physician is to be morally justified in using a placebo here, I suggest that she must be justified in believing that no reasonable further steps can be taken to ascertain the nature of the patient’s condition. In order to be justified in holding *this* belief, the physician must have formed this belief in accordance with epistemic standards set by the relevant professional body.

14. There have been some studies of patient attitudes toward placebo treatments. However, their results are not entirely uniform. For instance, Chen and Johnson suggest that their findings indicate that in many situations “. . . patients appear to be paternalistic in their attitudes to the deliberate use of placebo manipulations” (Chen and Johnson, 2009, 42). In contrast, Hull et al. claim that several of the responses in their study of patient attitude toward placebo use “. . . emphasize the value of physician honesty” (Hull et al., 2013, 3). See also Fässler et al. (2010) and Lynöe, Mattsson, and Sandlund (1993). Another problem with the cited studies is that none of them frame any of their questions pertaining to deceptive placebo use in a manner that makes it clear to respondents that the efficacy of placebos might depend on the patient being unaware that he is receiving a placebo. This is crucial, since the existing data thus do not tell us about attitudes to deceptive placebos of patients who hold the belief that the efficacy of placebos depends on their being administered deceptively.

15. The extent to which physicians currently observe these sorts of ethical principles is an empirical question that I cannot address here.

16. I thank an anonymous reviewer for pressing me on this point. It should be acknowledged, though, that, since placebos are inert, cases in which patients will suffer a bad reaction to them will be comparably rare.

17.
Foddy (2009a) argues for the stronger conclusion that physicians are warranted in presuming that patients would prefer to take a placebo that is the *best* available treatment and not merely the only treatment.

18. An anonymous reviewer suggested that deceptive placebo use could also undermine trust if patients were able to “see through” the physician’s attempts to deceive them. However, I believe that this too is unlikely. First, as I suggest in the above paragraph, the deception involved in deceptive placebo use is different in kind to the sorts of deception that we find problematic in medicine and that might justifiably undermine trust. Second, on a more practical point, it seems unlikely that patients would often see through their physician’s attempt to deceive them, since the deception involved in deceptive placebo use is not particularly complex; finally, as the UK data suggests, many GPs *already* seem to be administering placebos deceptively, and this has not yet led to a breakdown in doctor/patient relationships.
